# Neurofeedback in attention-deficit/hyperactivity disorder – different models, different ways of application

**DOI:** 10.3389/fnhum.2014.00846

**Published:** 2014-10-21

**Authors:** Holger Gevensleben, Gunther H. Moll, Aribert Rothenberger, Hartmut Heinrich

**Affiliations:** ^1^Child and Adolescent Psychiatry, University Medical Center GöttingenGöttingen, Germany; ^2^Department of Child and Adolescent Mental Health, University Hospital of ErlangenErlangen, Germany; ^3^Heckscher-KlinikumMünchen, Germany

**Keywords:** neurofeedback, attention-deficit/hyperactivity disorder (ADHD), model, learning, application, evaluation

## Abstract

In children with attention-deficit/hyperactivity disorder (ADHD), different neurofeedback (NF) protocols have been applied, with the most prominent differentiation between EEG frequency-band (e.g., theta/beta) training and training of slow cortical potentials (SCPs). However, beyond distinctions between such basic NF variables, there are also competing assumptions about mechanisms of action (e.g., acquisition of regulation capability, generalization to daily life behavior). In the present article, we provide a framework for NF models and suppose two hypothetical models, which we call “conditioning-and-repairing model” and “skill-acquisition model,” reflecting extreme poles within this framework. We argue that the underlying model has an impact not only on how NF is applied but also on the selection of evaluation strategies and suggest using evaluation strategies beyond beaten paths of pharmacological research. Reflecting available studies, we address to what extent different views are supported by empirical data. We hypothesize that different models may hold true depending on the processes and behaviors to be addressed by a certain NF protocol. For example, the skill-acquisition model is supported by recent findings as an adequate explanatory framework for the mechanisms of action of SCP training in ADHD. In conclusion, evaluation and interpretation of NF trials in ADHD should be based on the underlying model and the way training is applied, which, in turn, should be stated explicitly in study reports.

## INTRODUCTION

Overwhelming evidence exists for (1) the plasticity of the human brain, especially in childhood (Pascual-Leone et al., 2005), (2) distinct brain electrical patterns in cognitive and emotional processing (Banaschewski and Brandeis, 2007), and (3) the possibility to modulate brain electrical activity via neurofeedback (NF) in animals and humans (Banaschewski and Brandeis, 2007; Heinrich et al., 2007; Sherlin et al., 2011). Hence, there is growing interest in EEG-based NF as a treatment option for children with attention-deficit/hyperactivity disorder (ADHD) as documented for example by an increasing number of randomized controlled trials (RCTs) which have been conducted to study clinical efficacy and mechanisms of actions (for review see Arns et al., 2014; Gevensleben et al., 2014). However, diverging opinions exist how to interpret the results of the available studies regarding clinical efficacy (for example, Arns and Strehl, 2013 vs. Sonuga-Barke et al., 2013).

In a RCT of our group, NF comprised two “standard-protocols”: theta/beta training [aiming to decrease theta (4–8 Hz) activity and to increase beta (13–20 Hz) activity] and training of slow cortical potential (SCP; associated with the bidirectional regulation of cortical excitability). Both protocols were trained in separate blocks and paralleled regarding the setting and demands upon the participants. For NF, we obtained a larger reduction of the severity of ADHD symptoms (medium effect size) compared to a computerized attention skills training (“active control group”; Gevensleben et al., 2009a, 2010). Further, while linking brain electrical measures to the clinical outcome protocol-specific associations provided further evidence for the specificity of effects of theta/beta training and SCP training (Gevensleben et al., 2009b; Wangler et al., 2011).

In clinical practice, so-called QEEG (quantitative EEG)-based NF is also applied in ADHD. Before starting QEEG-based NF, multichannel-EEG is recorded and compared to a database of typically developing children. Frequency band and electrode location showing the greatest deviance from the “norm” are targeted during training. Related to this individualized approach, a randomized double-blind placebo-controlled trial was conducted by van Dongen-Boomsma et al. (2013). For pre-selected patients, mainly theta and sensorimotor rhythm (12–15 Hz) activity at frontal, central, or parietal leads were addressed in the training. Regarding the overall severity of ADHD symptoms, NF was not superior to the placebo (sham) training though a medium effect size for the symptom domain hyperactivity/impulsivity indicated some advantage for NF.

From the short descriptions of these two RCTs, it becomes apparent that different NF protocols and different evaluation strategies have been applied^1^. However, having a closer look beyond protocols and control conditions, it also turns out that there are different ways a NF training is realized depending on the different underlying model of action, i.e., assumptions regarding the underlying neuronal and psychological mechanisms as well as moderating and mediating factors affecting the effects of NF training (e.g., what are the mechanisms of learning and generalization in NF, how should supposed mechanisms underlying behavioral changes be addressed in the training?). So far, these aspects have not been considered adequately regarding NF in ADHD.

In the present article, we intend to provide a framework for defining NF models based on those above-mentioned aspects. To underline the relevance of the framework, which is suggested for theoretical and practical purposes, we reflect available studies and illustrate that different views are supported by empirical data.

## MECHANISMS AND APPLICATIONS – MODELS OF NF IN CHILDREN WITH ADHD

### COMPETING ASSUMPTIONS ABOUT NF

#### What is the indication for NF: repairing a neural dysfunction vs. strengthening resources/compensatory mechanisms on different levels

Application of NF in children with ADHD directly evolved from considerations about distinct neurophysiological dysfunctions (reviewed for example by Albrecht et al., submitted), encompassing different brain electrical activity parameters and electrode locations. Elevated theta/beta ratios in the resting EEG, reflecting reduced tonic cortical activation (Barry et al., 2003, 2009), and a reduced contingent negative variation (CNV; an event-related potential component associated with cognitive preparation) in cued attention tasks (Banaschewski and Brandeis, 2007), served as rationales to apply theta/beta training and SCP training, respectively.

Following a classical medical model of (psychiatric/neurodevelopmental) disorders, “repairing” the presumed cause (neurophysiological deficit) should “normalize” behavior: “The principle of NF is that over time, participants learn operant control of their EEG and change from an ‘abnormal’ state to one resembling that of typically developing children. This process is thought to eventually remediate the symptoms associated with ADHD” (Bakhshayesh et al., 2011, p. 482).

On the other hand, NF may simply be regarded as “a tool for enhancing specific cognitive or attentional states in certain situations” (Gevensleben et al., 2009a, p. 781), irrespective of presumed distinct neurophysiological deficits. The application of NF to improve “peak performances” in arts or sports is based on such an approach of “optimizing” rather than “repairing” (e.g., Landers et al., 1991; Egner and Gruzelier, 2003, for a review see Gruzelier, 2014b).

Nowadays, etiology of (psychiatric/neurodevelopmental) disorders is rather investigated on the basis of a bio-psycho-social model, considering the impact of different factors on different levels. Accordingly, regarding treatments, different areas of impact should also be taken into account. Therefore, NF does not necessarily need to address only a distinct neural dysfunction but may encompass (compensatory) mechanisms on different interacting levels, the strengthening of neural resources as well as changes of cognitive-behavioral and social variables^2^.

#### The effect of NF: is there a change of “EEG trait^3^” or a change in “EEG state”?

Particularly traditional models of NF in child and adolescent psychiatric disorders consider a stable change in the EEG signature (*“EEG trait”*) in terms of a durable change in protocol-specific EEG activity (Lubar and Shouse, 1976). Change of the “*EEG trait”* is typically assessed studying pre–post-changes in the *resting* EEG. Resting EEG in this case is considered to represent a kind of individual signature of the brain.

Others tend to expect an improved skill to change the “*EEG state”* in order to optimize performance temporarily (i.e., to improve attentional self-regulation; Heinrich et al., 2004). Regulation-skill refers to self-initiated effort of “activating and maintaining a state of cortical arousal” (Bakhshayesh et al., 2011) and is assessed during task performance. This perspective underlines the active part the subject plays in the allocation of attentional resources. From this point of view, changes after NF might not only be detectable by resting EEG assessment but should also be reflected in neurophysiological (and cognitive) patterns during task performance.

#### Neuro-regulation – implicit vs. explicit learning?

Concerning core mechanisms of NF, changes in within-session neuro-regulation (i.e., changes in EEG activity during treatment sessions) and improvements in neuro-regulation as the training proceeds are expected before changes in the clinical outcome result. Such systematic changes in EEG activity following positive reinforcement could be obtained in animals as well as in humans (e.g. Strehl, submitted). However, learning might evolve in an implicit (unconscious and automatic) and/or explicit (goal directed, controlled, and attention-demanding) way.

Implicit learning is defined as “the acquisition of knowledge that takes place largely independently of conscious attempts to learn and largely in the absence of explicit knowledge about what was acquired” (Reber, 1993, p. 5). Automatic processing and effortlessness of the procedure is postulated by some authors: “*Learning occurs as the child’s brain adjusts and interprets the cause-and-effect relationship between its own activity and the resultant video game responses*” (Steinberg and Othmer, 2004, p. 34); “w*hen the children and adolescents played the video game or watched the films, they produced brainwave activity that was ‘shaped’ toward more regulated performance*” (Duric et al., 2012, p. 3). Therefore, NF in children with ADHD might be considered an intervention “... *which trains the brain, via operant conditioning, to improve its regulation of itself...”* (Arnold et al., 2012, p. 410). Referring to voluntary control of circumscribed brain regions using real-time functional MRI, Birbaumer et al. (2013, p. 298) suggest that “*brain responses are learned, stored, and retained in a manner that is comparable to a motor skill, following the rules of implicit learning. In contrast to explicit learning, implicit learning and memory do not require conscious and effortful search.”*

At least it seems plausible that learning of neuro-regulation is enhanced by precise monitoring of the EEG signals being fed back, searching for a link between internal regulation and the mirrored neuronal signals, intentional building, and testing of cognitive strategies to shift generated EEG activity pattern in the required direction (for an overview of learning mechanisms in NF see Strehl, submitted). Therefore, controlled cognitive processes may also be involved in the acquisition of neuro-regulation capability (and generalization of self-regulation ability to daily life; see following section), suggesting rather an active role for the participant.

A further, often neglected notion is the superior cognitive level of expectations and attributions of patients: how do the participants perceive the training and participate in the exercises, what is the role of motivational, attributional, and personality factors for the course and outcome of the training (Meichenbaum, 1976)? Are these factors and associated underlying networks (e.g., mesocorticolimbic dopaminergic system related to motivational aspects) also modulated by the training?

#### Generalization – does it occur automatically or is special effort needed to achieve transfer into daily life?

Training effects should not be restricted to the environment where the NF training is conducted. NF strives for behavioral changes in daily life. If NF repairs an underlying neural dysfunction and/or learning happens automatically and unconsciously then generalization should occur automatically: “... *when brain behavior is normalized, the child’s behavior follows*” (Steinberg and Othmer, 2004, p. 35).

If NF relies on controlled learning and acquisition of skills and outcome depends on attributions and motivation, additional effort to transfer novel skills into daily life appears necessary in order to improve efficacy and clinical value.

### IMPLICATIONS FOR THE APPLICATION OF NF

The assumptions reflected in the previous section impact the way NF training is applied.

#### Indication – “Repairing” a neural dysfunction vs. strengthening neural resources

Assuming a distinct neurophysiological dysfunction to be addressed in NF training in children with ADHD has implications for the indication of NF. Primarily, subjects with a manifest neurophysiological dysfunction (e.g., enhanced theta and/or reduced beta activity) are expected to improve behavior after NF training addressing this particular dysfunction (e.g., Monastra et al., 2002). Consequently, in a trial proposal by a collaborative NF group only children with significantly enhanced theta/beta ratios will be included (Kerson and Collaborative Neurofeedback Group, 2013). Treatment solely targets the distinct neurophysiological dysfunction.

If, on the other hand, NF is expected to exert its effects via (compensatory) mechanisms on different (neurophysiological and cognitive-behavioral) levels pre-selection based on distinct neurophysiological profiles does not play an essential role. Room for improvement in self-regulation on the neurophysiological and cognitive-behavioral level provides rough indication criteria, hopefully in the future differentiated and optimized by knowledge about moderators of outcome (neurophysiological, cognitive, and social predictors of improvement such as distinct EEG parameters, personality variables, and supporting social conditions). Treatment focuses on neurophysiological functioning during the treatment sessions but also targets further variables on the cognitive-behavioral (self-efficacy, achievement motivation) and social level (social reinforcement), interacting with the achievement in neuro-regulation capability. Further effort (via cognitive-behavioral interventions such as education/instructions, positive/social reinforcement, transfer tasks/home work and parent/teacher counseling) is spent to ensure enhancement of general behavioral self-regulation capability (Gevensleben et al., 2012), i.e., the goal may be a personalized combination of machine-guided and trainer-guided learning.

#### Acquisition of (neuro-)regulation: mechanisms of learning, mechanisms of change

Mechanisms of the acquisition of neuro-regulation capability beyond basic operant mechanisms (reaction-consequence-contingency; positive reinforcement; for an overview see Sherlin et al., 2011) are not elucidated satisfactorily. Assumptions about the mechanisms of learning (e.g., how to achieve EEG changes during sessions) affect further aspects of the application of NF (e.g., via the attitude of the trainer, the introduction of the training, the level of ambition, and the instructions before and during treatment; Meichenbaum, 1976). If one expects NF to work in an automatic and unconscious manner, participants are instructed accordingly: “*the participant was instructed that the brightening of the movie screen and the audio clicks are good signs and that the learning process is mostly unconscious so no specific effort is needed*” (Logemann et al., 2010, p. 51). NF systems are considered to work autonomous, a “NF coach” to guide trainees is not required (e.g. Arnold et al., 2012).

In concurring approaches the need for active and effortful engagement is emphasized: “*Children were only advised to be attentive to the feedback and to find the most successful mental strategy to move the ball into the required goal. Because there is no unique cognitive strategy for the task, examples were given that have been shown to be successful in at least some children. Between runs, therapists asked the subjects to verbalize strategies and encouraged them to try new strategies or stick to the successful ones*” (Strehl et al., 2006, p. e1533).

#### How to assure generalization?

To assume that generalization of effects occurs automatically (via change of “EEG trait”) makes further efforts obsolete. Enduring and general change in significant EEG pattern after NF training should lead to enduring and general change in daily behavior automatically.

On the other hand, if NF is interpreted as a neuro-behavioral treatment aimed at developing skills not only for self-regulation of brain activity but also for general behavior in daily life, additional elements are introduced in the training (Heinrich et al., 2007). To support transfer into daily life, some authors established transfer trials where no contingent feedback is provided (see, e.g., Strehl et al., 2006) and force participants to practice regulation skills in daily life. Parents are instructed to spend support: “*the trainer encouraged the child to develop an appropriate strategy … to work out a plan how and where to use the strategy in daily life, discussed problems encountered with transfer and introduced a training diary.” “Parents were invited to participate at training sessions and to supervise transfer training with cards at home”* (Drechsler et al., 2007, p. 5).

### “CONDITIONING-AND-REPAIRING” vs. “SKILL-ACQUISITION” MODEL

**Table 1** summarizes and contrasts different assumptions concerning models and applications of NF underlying different NF approaches. Assumptions are contrasted by two hypothetical models. Both models are proposed only for didactic reasons and represent extreme poles of concurring assumptions. Models comprising assumptions from either side or combining elements from both sides (e.g., interaction of implicit and explicit learning processes) can be developed.

**Table 1:** Concurring assumptions and resulting ways of application regarding NF (in attention-deficit/hyperactivity disorder, ADHD).

	“Conditioning-and-repairing model”	“Skill-acquisition model”
**Assumptions**		
Indication	Specific neurophysiological deficit	No specific deficit
Mechanisms of learning (EEG regulation acquisition)	Automatic, unconscious (implicit) learning (operant conditioning of EEG pattern)	Controlled, effortful acquisition of regulation skills (explicit learning)
Significance of psychological and social variables and personality traits as moderators/mediators	Susceptibility to basic learning mechanisms (operant conditioning), no higher-order cognitive processes involved.	Effects moderated/mediated by cognitive-attributional variables; generalization of effects moderated by social support, positive reinforcement of target behavior
Effects of the treatment	Automatic change in EEG-trait (tonic change).	Change in EEG-state (phasic changes), acquisition of self-regulation skills, enhancement of neurophysiological functioning
**Ways of application**		
Instructions, acquisition of self-regulation	No active trainer, no specific instructions/no effort needed, passive participant	Active coaching, support in the search for regulation strategies, active participant, effort to enhance self-regulation skills
Generalization	Automatic transfer to daily life → no effort necessary to support generalization	Transfer-trials; tasks for generalization of effects (e.g., homework)
Setting	Unimodal treatment (Repairing the EEG deficit “normalizes” behavior.)	Module in a multimodal treatment, involvement of parents/teachers

The so called “*conditioning-and-repairing model*” encompasses a somehow more traditional view of NF and follows a mono-causal medical model as treatment targets a distinct causal deficit. Key assumptions are that NF repairs an initial neural dysfunction by implicit operant conditioning processes. Attenuation of this deficit leads to attenuation of the symptoms.

Alternatively the so called “*skill-acquisition model*” is based on a biopsychosocial model, taking different possible conditions and levels in the development and maintenance of symptoms into account. It underlines effortful, controlled (explicit) learning and the necessity to support generalization of acquired skills directly by cognitive-behavioral strategies. NF training targets self-regulation on a neurophysiological and a cognitive-behavioral level, both representing two sides of the same coin, targeted from both directions, on the neurophysiological and the cognitive-behavioral level. In contrast to the conditioning-and repairing-model, improved neuroregulation and clinical outcome (reduction of the severity of ADHD symptoms) are not necessarily strongly correlated.

#### Annotations about specificity of treatments

The distinction of specific vs. non-specific variables of a treatment also relies on the underlying model. Continuous monitoring of behavior, contingent feedback, and positive reinforcement might be considered powerful variables of NF. During NF, monitoring, feedback, and reinforcement impact the neurophysiological as well as the cognitive-behavioral level. In view of a conditioning-and-repairing model, reinforcement on the cognitive-behavioral level (praise by the trainer, pride about a good score, both leading to enhanced self-efficacy) constitutes an unspecific variable. On the background of a “skill acquisition model,” these are basic variables and essential prerequisites of further treatment variables (neuro-regulation).

## IMPLICATIONS FOR THE EVALUATION OF NF

### HOW TO EVALUATE EFFICACY OF NF? WHICH VARIABLES ACCOUNT FOR THE EFFICACY OF NF? WHICH VARIABLES SHOULD BE CONSIDERED “SPECIFIC” OR “UNSPECIFIC”? CAN THE FIDELITY OF THE NF TREATMENT BE ENSURED UNDER PLACEBO-CONTROL CONDITIONS?

There is no doubt that RCT are necessary to evaluate efficacy of NF in the treatment of children with ADHD. In pharmacological research, double-blind, placebo-controlled trials are considered the gold standard in the evaluation of efficacy. Concerning the mechanisms of action of pharmacological treatment, placebo conditions should allow a valid separation of specific from non-specific effects. Blindness to the treatment condition and placebo-control are meant to level the expectations of the participants about the treatments. This is reasonable in the evaluation of efficacy of treatments, if a treatment does not rely on the participant’s expectations and active engagement.

Larger effects of NF compared to placebo training would indicate efficacy and specificity of NF. Unfortunately, previous placebo-controlled trials found no superiority of NF in children with ADHD (see Vollebregt et al., 2014b)^4^. However, as stated by Vollebregt et al. (2014b, p. 02): “absence of evidence does not equate with evidence of absence.” If NF does not turn out to be superior to placebo training in certain trials (e.g., Logemann et al., 2010; van Dongen-Boomsma et al., 2013) different reasons come into account – first and foremost treatment fidelity. In NF trials confirming the null-hypothesis, it should be obligatory to analyze pre- and post-training EEG data and especially the course of regulation-data to ensure that the training was accompanied by corresponding changes in the resting EEG and, most important, that regulation capability evolved adequately in the NF group (and, if at all, increased to a smaller extent in the placebo group). If participants fail to acquire regulation capability during the treatment (as reported, e.g., in Vollebregt et al., 2014a related to the report of van Dongen-Boomsma et al., 2013), fidelity of the training must be considered seriously impaired and the most likely explanation for the results of hitherto existing placebo-controlled trials is that the key mechanism of NF, the operant learning to alter EEG patterns, was knocked out (for detailed comments to previous placebo-NF-trials, see Arns et al., 2014). Other aspects can also impair fidelity of the application of NF (Sherlin et al., 2011). In this manuscript, we primarily consider feasibility of placebo-controlled trials on the background of different NF models.

Following a “*conditioning and repairing model,*” placebo-controlled trials constitute a valid strategy for the evaluation of NF. The efficacy of the treatment is assumed to rely on changes of EEG patterns, automatically achieved by operant conditioning via NF by implicit learning mechanisms. Expectations of the participants carry no weight and no effort for further generalization of the treatment effects must be spent.

Following the “*skill acquisition model*” evaluation should follow criteria employed in the evaluation of cognitive-behavioral interventions. According to a “*skill acquisition model*” efficacy of NF treatment in ADHD does not (solely) rely on implicit and tonic changes in EEG but improved skills of self-regulation, acquired during treatment sessions and furthermore during transfer-tasks at home (Gevensleben et al., 2012) – and probably also touching other neuronal circuits than those primarily addressed by the feedback protocol. Variables like treatment credibility, outcome expectation, self-efficacy, achievement motivation, or locus of control are assumed to be basic moderators of treatment (Borkovec and Sibrava, 2005; Gevensleben et al., 2012). In other words, specific variables are thought to depend on those essential “unspecific” but basic variables. Participant’s estimation of practicing placebo training may impair treatment credibility, outcome expectation, self-efficacy, effort spent in skill acquisition, and transfer into daily life. Following the “*skill acquisition model,*” fidelity of the treatment may be seriously impaired in placebo-controlled NF trials ^5^.

Though no NF study in the fields of ADHD to date directly investigated the moderating effects of those basic variables, the results of latest placebo-controlled trials (as reported above) are in line with the aforementioned assumption.

Active control conditions may be preferable (e.g., computerized attention training, EMG biofeedback training including a feedback of artifacts derived from the EEG; Heinrich et al., 2007; Holtmann et al., 2014; Maurizio et al., 2014), paralleled with respect to the setting and the demands upon the participants as well as to the expectations and attributions. In addition, basic (“unspecific”) factors (e.g., expectations) can either be controlled for by using appropriate questionnaires (Kotchoubey et al., 2001; Gevensleben et al., 2009a) or could be systematically manipulated via instructions to assess their influence on treatment outcome (Goldberg et al., 1982; Holroyd et al., 1984).

A comparison of different NF protocols can be regarded as another evaluation strategy which may be applied irrespective of the underlying model. Larger clinical improvements for one NF protocol than another provides clear evidence for specific effects (e.g., Gevensleben et al., 2014). Moreover, distinct effects at the neurophysiological level (associated with the clinical outcome) may further indicate specificity of effects.

## EMPIRICAL EVIDENCE

In the “Mechanisms and Applications – Models of NF in Children with ADHD” section, we assembled concurring assumptions about the indication, mechanisms of change, and effects of NF. In the following we will highlight some empirical evidence concerning each of the above mentioned notions. Empirical evidence concerning certain aspects of NF is rare and contradictory. Most studies evaluated outcome of NF rather than treatment processes. Hence, valid data concerning prerequisites and predictors of outcome as well as mechanisms of change (learning, generalization) often is missing. We will focus on standard protocols of NF in children with ADHD (theta/beta and SCP training) and lend some findings from trials with healthy adults if indicated. Our aim is not to give an exhaustive review of the existing literature but to substantiate our aforementioned considerations and to elucidate the eligibility of the presented models of NF in order to encourage further research elucidating mechanisms of NF. A comprehensive overview concerning the empirical validation of NF in healthy adults is provided by Gruzelier (2014a,b,c).

### INDICATION FOR NF AND EFFECTS/RESULTS OF NF

#### What is the evidence for distinct neurophysiological deficits in ADHD?

An elevated theta/beta ratio (enhanced theta activity, reduced beta activity) has been considered a neurophysiological marker of children with ADHD (Snyder and Hall, 2006) and represents the background for the application of theta/beta-NF in children with ADHD. In the light of latest empirical findings, at least the general assumption of a neurophysiological deviation in case of an elevated theta/beta ratio at rest in children with ADHD is arguable. Recent studies (e.g., meta-analysis by Arns et al., 2012; Liechti et al., 2012) conclude that, at most, only a subgroup of children with ADHD exhibit this feature. Regarding an elevated theta/beta ratio as an indication criterion for theta/beta treatment therefore would limit the target population to only a small subgroup of children with ADHD.

On the other hand, Heinrich et al. (2014) reported inter alia increased theta activity in children with ADHD during an attentive state in a cognitive task though an increased theta/beta ratio only characterized children of the predominantly inattentive subtype of ADHD. Children of the combined type showed the largest deviation in the upper-theta/lower-alpha (5.5–10.5 Hz) range.

A reduced CNV has been reported in the major part of studies in children with ADHD (for review see Albrecht et al., submitted) though complexity (Bruckmann et al., 2012), age of the participants (Kratz et al., 2011), and aspects of comorbidity (Banaschewski et al., 2003) may affect results.

Generally it should be kept in mind that the mentioned “neuronal deficits” represent only some neuronal correlates of disturbed behavior but do not give a full explanation of the complex ADHD picture. Hence, the thinking of “just repairing” appears to be rather simplistic. Moreover, as ADHD is considered a clinically and pathophysiologically heterogeneous condition, it appears rather likely that a deviant neurophysiological pattern is not shared by all children with ADHD.

#### Does NF repair this neurophysiological deficit or strengthen compensatory mechanisms? Is there a change of “EEG trait” or a change in “EEG state”?

***Frequency band training.*** In children with ADHD reliable evidence indicating post-treatment protocol-specific lasting change in resting EEG (“trait”) is lacking. Several previous trials abstained from assessing pre–post-change of resting EEG after NF.

We found a decrease in theta activity (no change of beta activity) in the resting EEG after NF, irrespective of the treatment protocol (SCP- vs. theta/beta training; Gevensleben et al., 2009b). There is some evidence that enhanced theta activity predicts superior outcome after theta/beta training. After 18 sessions of theta/beta training, larger improvements were related to higher baseline theta activity, as well as to a larger reduction of theta activity, mainly at parietal-midline sites (Gevensleben et al., 2009b). These results would indicate that the “worst cases” (high baseline-theta) improve the most. So, this result may be considered in line with the assumption that the more the initial deficit is “repaired,” the more improvement in behavior can be observed.

On the other hand, it has to be considered that, during training, children practiced to get into an “active,” attentive state (in contrast to the resting condition) and that no effects were observed for beta activity and the theta/beta ratio, respectively, which were also targeted during training.

Monastra et al. (2002) reported a decrease of the theta/beta ratio after theta/beta training in children with ADHD characterized by a high baseline theta/beta ratio. Effect sizes of EEG changes as well as regarding behavioral measures in this study including pre-selected children with ADHD outperform all other controlled NF trials. However, among other differences, pre- and post-training EEG assessment encompassed several conditions (resting and active conditions) and might therefore also display enhanced regulation-skills, changing task-specific “EEG state” rather than general “EEG trait.”

In healthy adults, associations between distinct NF protocols and changes in the spectral topography of the resting EEG do not support the change of EEG trait notion (Egner et al., 2004) or are at least inconclusive (Gruzelier, 2014c). For example, Doppelmayr et al. (2009) obtained no significant increase of sensorimotor rhythm (SMR, 12–15 Hz) activity in the resting EEG after 25 units of SMR training, although there were solid increases of SMR amplitudes during training.

Evidence for protocol-specific general and lasting change in resting EEG activity (that we would consider a change of EEG trait) is inconclusive. However, sustainability of NF induced tonic resting EEG changes might depend on the number of treatment sessions. Maybe more sessions than conducted in previous trials would be necessary to achieve enduring change ^6^.

***SCP training.*** SCP training was associated with CNV effects in a number of trials in children with ADHD (Heinrich et al., 2004; Doehnert et al., 2008; Wangler et al., 2011) representing a change in the short-term mobilization of cortical resources reflecting a change in EEG state. It has to be noted that not children with an initially more reduced CNV (pronounced deficit) but those children with a higher CNV (less pronounced deficit) improved more after SCP training (Wangler et al., 2011). Thus, outcome of treatment may rather rely on the better access to basic neurophysiological resources: the more resources are available at baseline, the better the outcome of the NF treatment (“the best cases improve the most”).

All in all, there is valid evidence that SCP training attenuates an initial deficit in regulation of cortical excitability in children with ADHD. There is also evidence that a link between the neurophysiological and the cognitive-behavioral level contributed to improved clinical outcome.

### ACQUISITION OF NEURO-REGULATION CAPABILITY – LEARNING DURING NF SESSIONS

#### Implicit or explicit learning of neuro-regulation?

Investigation of implicit learning mechanisms relies on dissociation paradigms to prevent awareness of the learning process. No serious attempt has been practice in neuro-regulation until now in order to assess, in how far neuro-regulation might evolve implicitly in first and foremost.

In a recent summary of their efforts to elucidate the acquisition of regulation capability Birbaumer et al. (2013) “...*propose that self-regulation of brain activity is akin to skill learning and thus may depend on an intact subcortical motor system...*” as well as “... *that brain-self-regulation need not be an explicit and conscious process...*” (Birbaumer et al., 2013, p. 295). Concerning regulation capability, activity of the basal ganglia and cortical motor structures appeared to play a significant role in the differentiation of good against poor learners (Hinterberger et al., 2005; Birbaumer et al., 2013) and the deletion of striatal NMDA receptors in rodents eliminated the ability to develop neuro-regulation skills (Koralek et al., 2012). This finding indicates that acquisition of neuro-regulation relies on similar neuronal structures and might develop similar to motor skill acquisition (though it has to be kept in mind that cognitive and motor circuits are functionally segregated within these structures; Alexander et al., 1986).

However, the forced acquisition of neuro-regulation skills during scientific trials or clinical applications in humans and even in rodents proceeds not implicitly (out of awareness) but intentional and goal-directed (Kübler and Birbaumer, 2008; Koralek et al., 2012). Philippens and Vanwersch (2010) trained marmoset monkeys to voluntary control their SMR brain activity (11–14 Hz) and omitted the reinforcement after a successful trial: “*it was clearly seen that this monkey was expecting a reward immediately after the successful EEG pattern. This indicates that the monkey was aware that his mood or behavior expressed by the brain activity was related to the reward*” (Philippens and Vanwersch, 2010, p. 330). Furthermore, Ninaus et al. (2013) obtained that regulation effort during NF is accompanied by activity in frontoparietal and cingulo-opercular networks involved in cognitive control.

Therefore, although acquisition of neuro-regulation encompasses mechanisms of procedural learning, there is no clear evidence until now, that neuro-regulation primarily results from implicit learning (automatic, out of conscience, not goal-directed).

Acquisition of neuro-regulation seems to depend on attention (Daum et al., 1993) and motivation (Kathner et al., 2013), is distracted by parallel/concurring information (Johnson et al., 2012), and influenced by affect, attribution, and personality (Hardman et al., 1997; Witte et al., 2013; Kotzias, unpublished). Furthermore, there is a large variability in the success of acquiring neuro-regulation between subjects with a significant rate of non-learners (Drechsler et al., 2007). These findings do not support the notion of pure implicit learning of neuro-regulation. However, sometimes explicit, controlled processing may disrupt implicit learning, e.g. if exceeding verbalization induces an explicit learning mode in the performance of a (procedural) task which is not suitable for predominant explicit processing (Reber, 1993; Sun et al., 2005; Drechsler et al., 2007)

Due to the fact that implicit and explicit processes usually interact in skill learning (Sun et al., 2005; Goujon et al., 2014), it is reasonable to assume that different learning mechanisms interact in neuro-regulation. Acquisition of skills in complex tasks is considered a “*vital interplay that occurs between automatic and controlled processes throughout skill development*” (Shebilske et al., 1999, p. 402). The acquisition of (motor) skills evolves at different stages, initially requiring controlled and effortful processing (e.g. trial and error) developing to more automated and effortless skills (e.g. from declarative to procedural knowledge, Anderson, 1983). In clinical settings (as for the application of NF in children with ADHD), the acquisition of neuro-regulation is a goal directed, self-referential procedural learning process, presumably encompassing interacting implicit as well as explicit learning mechanisms. However, controlled experimental trials disentangling learning mechanisms, including analysis of the relevant cortical and subcortical neural structures, are still outstanding.

### GENERALIZATION

#### Automatic generalization

After a single session of NF (voluntary alpha-attenuation), Ros et al. (2013) obtained an enduring increase of salience network activity (at least 30 min after treatment) in healthy adults reflecting a neuroplastic effect. Furthermore, a single session of NF facilitated performance in a procedural learning task, also without explicit instruction to transfer a regulation “strategy” to the upcoming task (Ros et al., 2014).

In Hoedlmoser et al. (2008) and Schabus et al. (2014), 10 sessions of SMR enhancement led to enhanced expression of 12–15 Hz spindle oscillations during sleep and improved sleep quality, indicating even longer automatic changes in EEG activity associated with a better outcome. However, this trials were goal-directed (reducing sleep problems) and did not exclude that participants transferred strategies from treatment on their own effort.

#### Effortful transfer

On the other hand, the finding of Schafer and Moore (2011) obtained in monkeys who gained voluntary control over the activity of neurons within the frontal eye field indicates the necessity of an explicit transfer to take place since, after training, selective attention correlated only with voluntary fluctuations of frontal eye field activity.

Concerning ADHD, the outcome of SCP training may differ depending on whether transfer tasks rather address attentional or motor aspects. In several studies, SCP training induced comparable reductions of inattentive and hyperactive/impulsive behavior (e.g., Leins et al., 2006; Gevensleben et al., 2009a) or larger effects regarding inattention (Drechsler et al., 2007). However, the same SCP protocol only had a significant effect on hyperactivity/impulsivity but not inattention in our recent study (Gevensleben et al., 2014) investigating ADHD-related behavior in children with tic disorders. Application differed from previous ADHD trials with regard to treatment goals (improvement of motor inhibition in tic disorders), instructions, and transfer tasks/homework, probably accounting for the differential outcome pattern. It may be inferred that SCP regulation builds the basis for behavioral change and transfer tasks guide the direction.

## CONCLUSION AND IMPLICATIONS

There is strong evidence for the efficacy and specificity of certain NF approaches in ADHD, particularly SCP training applied as a neuro-behavioral treatment. Evidence results from trials using active control conditions, comparing different NF protocols and also by taking changes on the neurophysiological level into account. Hence, we argue that the guiding question today is, how to optimally use NF techniques to enhance efficacy of NF and how to optimize training for a certain participant (“personalized medicine”).

In the present article, we outlined that different models exist how NF may work (with the “conditioning-and-repairing model” and the “skill-acquisition model” representing two extreme poles) and that the underlying model unfolds implications for the application of the training as well as for the evaluation design of a RCT. These aspects may contribute to the divergent findings and interpretations regarding NF in ADHD. We recommend the following points for (future) NF trials in ADHD:

– As long as there is no detailed knowledge about the mechanisms of NF (in circumscribed fields of application) the assumptions about the mechanisms on which the application of NF is based shall be expatiated according to the framework proposed.
– It has to be checked that potential operators and moderators of efficacy are not attenuated by the design of the trial or the application of the NF protocol.
– Evaluation and interpretation of NF trials shall be based on the underlying model and the way training is applied.

Besides these aspects related to our framework, it is important that the application of NF follows the principles of learning theory (Sherlin et al., 2011). Moreover, particularly if the NF approach does not turn out to be superior to a control condition it is essential to document treatment fidelity in the way that successful neuro-regulation actually took place.

Reflecting the available literature, we suppose that

– NF is indicated whenever self-regulation ability should be enhanced and there is valid knowledge about neurophysiological target patterns.
– The acquisition of regulation capabilities advances in a goal-directed manner with implicit and explicit learning mechanisms interacting closely.
– Learning of neuro-regulation does not solely rely on neurophysiological preconditions but is significantly moderated by attributions, personality, and motivational factors and relies on personal effort.
– exhaustive rehearsal presumably leads to improved and automated regulation skills accompanied by changes in functional and structural brain “*trait”* in the long run.

Concerning the aspect of generalization, empirical findings indicate that different models may be valid depending on the NF protocol and mechanisms to be addressed by the training. We hypothesize:

– Distinct and circumscribed bottom-up mechanisms are enhanced by improved neurophysiological functioning alone (e.g., related to encoding or procedural learning in an experimental task).
– Complex attentional and social behaviors (encompassing different top–down and bottom–up mechanisms) rely to a larger extent on self-regulation skills and will not change to a clinically significant level due to distinct neurophysiological changes alone but have to be addressed on different levels. Neurophysiological changes must spread out beyond NF-trained neuronal circuits and be accompanied by changes in cognitive-behavioral patterns to achieve enhanced self-regulation in complex environments.

We are aware that evidence for these propositions is weak, However, they may serve as a clue for future studies that should target possible moderators (e.g., neurophysiological profile, comorbidity, social support, treatment setting) and mediators of change (e.g., neuro-regulation, changes in attributions, and behavioral skills) as well as obligatory vs. optional variables of a specific NF approach and model, respectively.

The scope should be widened from outcome to process evaluation to study an interplay of variables on different levels. This may not only comprise behavioral (e.g., severity of ADHD core symptoms and associated domains) and neurophysiological factors (neuro-regulation data over the course of the training; brain electrical activity at rest and during task performance in the lab) but also psychological and environmental aspects. In this context, we suggest to consider appropriate evaluation scales and to manipulate factors systematically as part of the research protocol (e.g., enhancing or diminishing treatment credibility or self-efficacy by specific instructions).

Conducting such studies would allow to fill gaps in current models of NF in ADHD gradually and to judge which model is suitable for which application (under which conditions).

## Conflict of Interest Statement

The authors declare that the research was conducted in the absence of any commercial or financial relationships that could be construed as a potential conflict of interest.

## Footnotes

1 There are also significant differences in the implementation of feedback protocols concerning aspects like fixed vs. variable thresholds, discrete vs. continuous feedback, discussed elaborately by Sherlin et al. (2011), Strehl (submitted).

2 Changes of cognitive-behavioral and social variables are also reflected in changes of underlying neural networks.

3 As “trait” we understand individual EEG patterns considered stable over time and situations, usually measured in resting conditions and representing an idiosyncratic EEG signature of a person. As “state” we define EEG activity based on the EEG trait, triggered by distinct situations and assessed during an active condition (typically during task performance).

4 In other areas, placebo-controlled designs were applied successfully (Gruzelier, 2014c).

5 Ethical aspects also argue against the use of a placebo condition (Gevensleben et al., 2014).

6 Increases in gamma activity in the resting EEG of Buddhist practitioners doing meditation training for 10.000–50.000 h over 15–40 years (Lutz et al., 2004) may reflect an extreme example.

## References

[B1] Alexander G. E. Delong M. R. Strick P. L. (1986). Parallel organization of functionally segregated circuits linking basal ganglia and cortex. *Annu. Rev. Neurosci.* 9 357–381. 10.1146/annurev.ne.09.030186.002041 3085570

[B2] Anderson J. R. (1983). *The Architecture of Cognition.* Cambridge, MA: Harvard University Press

[B3] Arnold L. E. Lofthouse N. Hersch S. Pan X. Hurt E. Bates B. (2012). EEG neurofeedback for ADHD: double-blind sham-controlled randomized pilot feasibility trial. *J. Atten. Disord.* 17 410–419. 10.1177/1087054712446173 22617866 PMC3939717

[B4] Arns M. Conners C. K. Kraemer H. C. (2012). A decade of EEG Theta/Beta ratio research in ADHD: a meta-analysis. *J. Atten. Disord.* 17 374–383. 10.1177/1087054712460087 23086616

[B5] Arns M. Heinrich H. Strehl U. (2014). Evaluation of neurofeedback in ADHD: the long and winding road. *Biol. Psychol.* 95 108–115. 10.1016/j.biopsycho.2013.11.013 24321363

[B6] Arns M. Strehl U. (2013). Evidence for efficacy of neurofeedback in ADHD? *Am. J. Psychiatry* 170 799–800. 10.1176/appi.ajp.2013.13020208 23820843

[B7] Bakhshayesh A. R. Hansch S. Wyschkon A. Rezai M. J. Esser G. (2011). Neurofeedback in ADHD: a single-blind randomized controlled trial. *Eur. Child Adolesc. Psychiatry* 20 481–491. 10.1007/s00787-011-0208-y 21842168

[B8] Banaschewski T. Brandeis D. (2007). Annotation: what electrical brain activity tells us about brain function that other techniques cannot tell us – a child psychiatric perspective. *J. Child Psychol. Psychiatry* 48 415–435. 10.1111/j.1469-7610.2006.01681.x 17501723

[B9] Banaschewski T. Brandeis D. Heinrich H. Albrecht B. Brunner E. Rothenberger A. (2003). Association of ADHD and conduct disorder–brain electrical evidence for the existence of a distinct subtype. *J. Child Psychol. Psychiatry* 44 356–376. 10.1111/1469-7610.00127 12635966

[B10] Barry R. J. Clarke A. R. Johnstone S. J. (2003). A review of electrophysiology in attention-deficit/hyperactivity disorder: I. Qualitative and quantitative electroencephalography. *Clin. Neurophysiol.* 114 171–183. 10.1016/S1388-2457(02)00362-0 12559224

[B11] Barry R. J. Clarke A. R. Johnstone S. J. Mccarthy R. Selikowitz M. (2009). Electroencephalogram theta/beta ratio and arousal in attention-deficit/hyperactivity disorder: evidence of independent processes. *Biol. Psychiatry* 66 398–401. 10.1016/j.biopsych.2009.04.027 19500774

[B12] Birbaumer N. Ruiz S. Sitaram R. (2013). Learned regulation of brain metabolism. *Trends Cogn. Sci.* 17 295–302. 10.1016/j.tics.2013.04.009 23664452

[B13] Borkovec T. D. Sibrava N. J. (2005). Problems with the use of placebo conditions in psychotherapy research, suggested alternatives, and some strategies for the pursuit of the placebo phenomenon. *J. Clin. Psychol.* 61 805–818. 10.1002/jclp.20127 15827991

[B14] Bruckmann S. Hauk D. Roessner V. Resch F. Freitag C. M. Kammer T. (2012). Cortical inhibition in attention deficit hyperactivity disorder: new insights from the electroencephalographic response to transcranial magnetic stimulation. *Brain* 135 2215–2230. 10.1093/brain/aws071 22492560

[B15] Daum I. Rockstroh B. Birbaumer N. Elbert T. Canavan A. Lutzenberger W. (1993). Behavioural treatment of slow cortical potentials in intractable epilepsy: neuropsychological predictors of outcome. *J. Neurol. Neurosurg. Psychiatry* 56 94–97. 10.1136/jnnp.56.1.94 8429329 PMC1014773

[B16] Doehnert M. Brandeis D. Straub M. Steinhausen H. C. Drechsler R. (2008). Slow cortical potential neurofeedback in attention deficit hyperactivity disorder: is there neurophysiological evidence for specific effects? *J. Neural Transm.* 115 1445–1456. 10.1007/s00702-008-0104-x 18762860

[B17] Doppelmayr M. Weber E. Hoedlmoser K. Klimesch W. (2009). Effects of SMR feedback on the EEG amplitude. *Hum. Cogn. Neurophysiol.* 2 21–32

[B18] Drechsler R. Straub M. Doehnert M. Heinrich H. Steinhausen H. C. Brandeis D. (2007). Controlled evaluation of a neurofeedback training of slow cortical potentials in children with Attention Deficit/Hyperactivity Disorder (ADHD). *Behav. Brain Funct.* 3 35. 10.1186/1744-9081-3-35 PMC1988816 17655749

[B19] Duric N. S. Assmus J. Gundersen D. Elgen I. B. (2012). Neurofeedback for the treatment of children and adolescents with ADHD: a randomized and controlled clinical trial using parental reports. *BMC Psychiatry* 12:107. 10.1186/1471-244X-12-107 PMC3441233 22877086

[B20] Egner T. Gruzelier J. H. (2003). Ecological validity of neurofeedback: modulation of slow wave EEG enhances musical performance. *Neuroreport* 14 1221–1224. 10.1097/00001756-200307010-00006 12824763

[B21] Egner T. Zech T. F. Gruzelier J. H. (2004). The effects of neurofeedback training on the spectral topography of the electroencephalogram. *Clin. Neurophysiol.* 115 2452–2460. 10.1016/j.clinph.2004.05.033 15465432

[B22] Gevensleben H. Holl B. Albrecht B. Schlamp D. Kratz O. Studer P. (2010). Neurofeedback training in children with ADHD: 6-month follow-up of a randomised controlled trial. *Eur. Child Adolesc. Psychiatry* 19 715–724. 10.1007/s00787-010-0109-105. 20499120 PMC3128749

[B23] Gevensleben H. Holl B. Albrecht B. Vogel C. Schlamp D. Kratz O. (2009a). Is neurofeedback an efficacious treatment for ADHD? A randomised controlled clinical trial. *J. Child Psychol. Psychiatry* 50 780–789. 10.1111/j.1469-7610.2008.02033.x 19207632

[B24] Gevensleben H. Holl B. Albrecht B. Schlamp D. Kratz O. Studer P. (2009b). Distinct EEG effects related to neurofeedback training in children with ADHD: a randomized controlled trial. *Int. J. Psychophysiol.* 74 149–157. 10.1016/j.ijpsycho.2009.08.005 19712709

[B25] Gevensleben H. Kleemeyer M. Studer P. Flaig-Röhr A. Moll G. H. Rothenberger A. (2014). Neurofeedback in ADHD: further pieces of the puzzle. *Brain Topogr.* 27 20–32. 10.1007/s10548-013-0285-y 23563906

[B26] Gevensleben H. Rothenberger A. Moll G. H. Heinrich H. (2012). Neurofeedback in children with ADHD: validation and challenges. *Exp. Rev. Neurother.* 12 447–460. 10.1586/ern.12.22 22449216

[B27] Goldberg J. Weller L. Blittner M. (1982). Cognitive self-control factors in EMG biofeedback. *Biofeedback Self Regul.* 7 545–551. 10.1007/BF00998893 7165785

[B28] Goujon A. Didierjean A. Poulet S. (2014). The emergence of explicit knowledge from implicit learning. *Mem. Cogn.* 42 225–236. 10.3758/s13421-013-0355-0 24052189

[B29] Gruzelier J. H. (2014a). EEG-neurofeedback for optimising performance. I: a review of cognitive and affective outcome in healthy participants. *Neurosci. Biobehav. Rev.* 44 124–141. 10.1016/j.neubiorev.2013.09.015 24125857

[B30] Gruzelier J. H. (2014b). EEG-neurofeedback for optimising performance. II: creativity, the performing arts and ecological validity. *Neurosci. Biobehav. Rev.* 44 142–158. 10.1016/j.neubiorev.2013.11.004 24239853

[B31] Gruzelier J. H. (2014c). EEG-neurofeedback for optimising performance III: a review of methodological and theoretical considerations. *Neurosci. Biobehav. Rev.* 44 159–182. 10.1016/j.neubiorev.2014.03.015 24690579

[B32] Hardman E. Gruzelier J. Cheesman K. Jones C. Liddiard D. Schleichert H. (1997). Frontal interhemispheric asymmetry: self regulation and individual differences in humans. *Neurosci. Lett.* 221 117–120. 10.1016/S0304-3940(96)13303-6 9121678

[B33] Heinrich H. Busch K. Studer P. Erben K. Moll G. H. Kratz O. (2014). EEG spectral analysis of attention in ADHD: implications for neurofeedback training? *Front. Hum. Neurosci.* 8:611. 10.3389/fnhum.2014.00611 PMC4139738 25191248

[B34] Heinrich H. Gevensleben H. Freisleder F. J. Moll G. H. Rothenberger A. (2004). Training of slow cortical potentials in attention-deficit/hyperactivity disorder: evidence for positive behavioral and neurophysiological effects. *Biol. Psychiatry* 55 772–775. 10.1016/j.biopsych.2003.11.013 15039008

[B35] Heinrich H. Gevensleben H. Strehl U. (2007). Annotation: neurofeedback – train your brain to train behaviour. *J. Child Psychol. Psychiatry* 48 3–16. 10.1111/j.1469-7610.2006.01665.x 17244266

[B36] Hinterberger T. Veit R. Wilhelm B. Weiskopf N. Vatine J. J. Birbaumer N. (2005). Neuronal mechanisms underlying control of a brain-computer interface. *Eur. J. Neurosci.* 21 3169–3181. 10.1111/j.1460-9568.2005.04092.x 15978025

[B37] Hoedlmoser K. Pecherstorfer T. Gruber G. Anderer P. Doppelmayr M. Klimesch W. (2008). Instrumental conditioning of human sensorimotor rhythm (12-15 Hz) and its impact on sleep as well as declarative learning. *Sleep* 31 1401–1408 18853937 PMC2572745

[B38] Holtmann M. Pniewski B. Wachtlin D. Wörz S. Strehl U. (2014). Neurofeedback in children with attention-deficit/hyperactivity disorder (ADHD) – a controlled multicenter study of a non-pharmacological treatment approach. *BMC Pediatr.* 14:202. 10.1186/1471-2431-14-202 PMC4134464 25123917

[B39] Holroyd K. A. Penzien D. B. Hursey K. G. Tobin D. L. Rogers L. Holm J. E. (1984). Change mechanisms in EMG biofeedback training: cognitive changes underlying improvements in tension headache. *J. Consult. Clin. Psychol.* 52 1039–1053. 10.1037/0022-006X.52.6.1039 6520274

[B40] Johnson K. A. Hartwell K. Lematty T. Borckardt J. Morgan P. S. Govindarajan K. (2012). Intermittent “real-time” fMRI feedback is superior to continuous presentation for a motor imagery task: a pilot study. *J. Neuroimaging* 22 58–66. 10.1111/j.1552-6569.2010.00529.x 20977537 PMC3070198

[B41] Kathner I. Ruf C. A. Pasqualotto E. Braun C. Birbaumer N. Halder S. (2013). A portable auditory P300 brain-computer interface with directional cues. *Clin. Neurophysiol.* 124 327–338. 10.1016/j.clinph.2012.08.006 22959257

[B42] Kerson C. Collaborative Neurofeedback Group. (2013). A proposed multisite double-blind randomized clinical trial of neurofeedback for ADHD: need, rationale, and strategy. *J. Atten. Disord.* 17 420–436. 10.1177/1087054713482580 23590978

[B43] Koralek A. C. Jin X. Long J. D. II Costa R. M. Carmena J. M. (2012). Corticostriatal plasticity is necessary for learning intentional neuroprosthetic skills. *Nature* 483 331–335. 10.1038/nature10845 22388818 PMC3477868

[B44] Kotchoubey B. Strehl U. Uhlmann C. Holzapfel S. Konig M. Froscher W. (2001). Modification of slow cortical potentials in patients with refractory epilepsy: a controlled outcome study. *Epilepsia* 42 406–416. 10.1046/j.1528-1157.2001.22200.x 11442161

[B45] Kratz O. Studer P. Malcherek S. Erbe K. Moll G. H. Heinrich H. (2011). Attentional processes in children with ADHD: an event-related potential study using the attention network test. *Int. J. Psychophysiol.* 81 82–90. 10.1016/j.ijpsycho.2011.05.008 21641942

[B46] Kübler A. Birbaumer N. (2008). Brain-computer interfaces and communication in paralysis: extinction of goal directed thinking in completely paralysed patients? *Clin. Neurophysiol.* 119 2658–2666. 10.1016/j.clinph.2008.06.019 18824406 PMC2644824

[B47] Landers D. M. Petruzzello S. J. Salazar W. Crews D. J. Kubitz K. A. Gannon T. L. (1991). The influence of electrocortical biofeedback on performance in pre-elite archers. *Med. Sci. Sports Exerc.* 23 123–129. 10.1249/00005768-199101000-00018 1997806

[B48] Leins U. Hinterberger T. Kaller S. Schober F. Weber C. Strehl U. (2006). [Neurofeedback for children with ADHD: a comparison of SCP- and theta/beta-protocols]. *Prax. Kinderpsychol. Kinderpsychiatr.* 55 384–407 16869483

[B49] Liechti M. D. Valko L. Muller U. C. Dohnert M. Drechsler R. Steinhausen H. C. (2012). Diagnostic value of resting electroencephalogram in attention-deficit/hyperactivity disorder across the lifespan. *Brain Topogr.* 26 135–151. 10.1007/s10548-012-0258-6 23053601

[B50] Logemann H. N. Lansbergen M. M. Van Os T. W. Bocker K. B. Kenemans J. L. (2010). The effectiveness of EEG-feedback on attention, impulsivity and EEG: a sham feedback controlled study. *Neurosci. Lett.* 479 49–53. 10.1016/j.neulet.2010.05.026 20478360

[B51] Lubar J. F. Shouse M. N. (1976). EEG and behavioral changes in a hyperkinetic child concurrent with training of the sensorimotor rhythm (SMR): a preliminary report. *Biofeedback Self Regul.* 1 293–306. 10.1007/BF01001170 990355

[B52] Lutz A. Greischar L. Rawlings N. Davidson R. J. (2004). Long-term meditators self- induce high-amplitude gamma synchrony during mental practice. *Proc. Natl. Acad. Sci. U.S.A.* 101 16369–16373. 10.1073/pnas.0407401101 15534199 PMC526201

[B53] Maurizio S. Liechti M. D. Heinrich H. Jancke L. Steinhausen H. C. Walitza S. (2014). Comparing tomographic EEG neurofeedback and EMG biofeedback in children with attention-deficit/hyperactivity disorder. *Biol. Psychol.* 95 31–44. 10.1016/j.biopsycho.2013.10.008 24211870

[B54] Meichenbaum D. (1976). Cognitive factors in biofeedback therapy. *Biofeedback Self Regul.* 1 201–216. 10.1007/BF00998587 990349

[B55] Monastra V. J. Monastra D. M. George S. (2002). The effects of stimulant therapy, EEG biofeedback, and parenting style on the primary symptoms of attention-deficit/hyperactivity disorder. *Appl. Psychophysiol. Biofeedback* 27 231–249. 10.1023/A:1021018700609 12557451

[B56] Ninaus M. Kober S. E. Witte M. Koschutnig K. Stangl M. Neuper C. (2013). Neural substrates of cognitive control under the belief of getting neurofeedback training. *Front. Hum. Neurosci.* 7:914. 10.3389/fnhum.2013.00914 PMC3872730 24421765

[B57] Pascual-Leone A. Amedi A. Fregni F. Merabet L. B. (2005). The plastic human brain cortex. *Annu. Rev. Neurosci.* 28 377–401. 10.1146/annurev.neuro.27.070203.144216 16022601

[B58] Philippens I. H. Vanwersch R. A. (2010). Neurofeedback training on sensorimotor rhythm in marmoset monkeys. *Neuroreport* 21 328–332. 10.1097/WNR.0b013e3283360ba8 20186109

[B59] Reber A. S. (1993). *Implicit Learning and Tacit Knowledge: An Essay on the Cognitive Unconscious*. New York: Oxford University Press

[B60] Ros T. Munneke M. A. Parkinson L. A. Gruzelier J. H. (2014). Neurofeedback facilitation of implicit motor learning. *Biol. Psychol.* 95 54–58. 10.1016/j.biopsycho.2013.04.013 23702458

[B61] Ros T. Theberge J. Frewen P. A. Kluetsch R. Densmore M. Calhoun V. D. (2013). Mind over chatter: plastic up-regulation of the fMRI salience network directly after EEG neurofeedback. *Neuroimage* 65 324–335. 10.1016/j.neuroimage.2012.09.046 23022326 PMC5051955

[B62] Schabus M. Heib D. P. Lechinger J. Griessenberger H. Klimesch W. Pawlizki A. (2014). Enhancing sleep quality and memory in insomnia using instrumental sensorimotor rhythm conditioning. *Biol. Psychol.* 95 126–134. 10.1016/j.biopsycho.2013.02.020 23548378

[B63] Schafer R. J. Moore T. (2011). Selective attention from voluntary control of neurons in prefrontal cortex. *Science* 332 1568–1571. 10.1126/science.1199892 21617042 PMC3371378

[B64] Shebilske W. Goettl B. Regian J. W. (1999). “Executive control and automatic processes as complex skills develop in laboratory and applied settings,” in *Attention and Performance XVII: Cognitive Regulation of Performance: Interaction of Theory and Application* eds Gopher D. Koriat A. (Cambridge, MA: MIT Press) 401–432

[B65] Sherlin L. A. Arns M. Lubar J. Heinrich H. Kersoni C. Strehl U. (2011). Neurofeedback and basic learning theory: implications for research and practice. *Neurotherapy* 15 292–304. 10.1080/10874208.2011.623089

[B66] Snyder S. M. Hall J. R. (2006). A meta-analysis of quantitative EEG power associated with attention-deficit hyperactivity disorder. *J. Clin. Neurophysiol.* 23 440–455. 10.1097/01.wnp.0000221363.12503.78 17016156

[B67] Sonuga-Barke E. Brandeis D. Cortese S. Daley D. Danckaerts M. Döpfner M. (2013). Response to Chronis-Tuscano et al and Arns and Strehl. *Am. J. Psychiatry* 170 800–802. 10.1176/appi.ajp.2013.13020208r 23820834

[B68] Steinberg M. Othmer S. (2004). *ADD: The 20-Hour Solution.* Bandon, OR: Robert Reed Publishers

[B69] Strehl U. Leins U. Goth G. Klinger C. Hinterberger T. Birbaumer N. (2006). Self-regulation of slow cortical potentials: a new treatment for children with attention-deficit/hyperactivity disorder. *Pediatrics* 118 e1530–e1540. 10.1542/peds.2005-2478 17060480

[B70] Sun R. Slusarz P. Terry C. (2005). The interaction of the explicit and the implicit in skill learning: a dual-process approach. *Psychol. Rev.* 112 159–192. 10.1037/0033-295X.112.1.159 15631592

[B71] van Dongen-Boomsma M. Vollebregt M. A. Slaats-Willemse D. Buitelaar J. K. (2013). A randomized placebo-controlled trial of electroencephalographic (EEG) neurofeedback in children with attention-deficit/hyperactivity disorder. *J. Clin. Psychiatry* 74 821–827. 10.4088/JCP.12m08321 24021501

[B72] Vollebregt M. A. Van Dongen-Boomsma M. Buitelaar J. K. Slaats-Willemse D. (2014a). Does EEG-neurofeedback improve neurocognitive functioning in children with attention-deficit/hyperactivity disorder? A systematic review and a double-blind placebo-controlled study. *J. Child Psychol. Psychiatry* 55 460–472. 10.1111/jcpp.12143 24168522

[B73] Vollebregt M. A. Van Dongen-Boomsma M. Slaats-Willemse D. Buitelaar J. K. (2014b). What future research should bring to help resolving the debate about the efficacy of EEG-neurofeedback in children with ADHD. *Front. Hum. Neurosci.* 8:321. 10.3389/fnhum.2014.00321 PMC4030169 24860487

[B74] Wangler S. Gevensleben H. Albrecht B. Studer P. Rothenberger A. Moll G. H. (2011). Neurofeedback in children with ADHD: specific event-related potential findings of a randomized controlled trial. *Clin. Neurophysiol.* 122 942–950. 10.1016/j.clinph.2010.06.036 20843737

[B75] Witte M. Kober S. E. Ninaus M. Neuper C. Wood G. (2013). Control beliefs can predict the ability to up-regulate sensorimotor rhythm during neurofeedback training. *Front. Hum. Neurosci.* 7:478. 10.3389/fnhum.2013.00478 PMC3744034 23966933

